# Leveraging SARS-CoV-2 Main Protease (M^pro^) for COVID-19 Mitigation with Selenium-Based Inhibitors

**DOI:** 10.3390/ijms25020971

**Published:** 2024-01-12

**Authors:** Viviana De Luca, Andrea Angeli, Alessio Nocentini, Paola Gratteri, Silvia Pratesi, Damiano Tanini, Vincenzo Carginale, Antonella Capperucci, Claudiu T. Supuran, Clemente Capasso

**Affiliations:** 1 Department of Biology, Agriculture and Food Sciences, National Research Council (CNR), Institute of Biosciences and Bioresources, 80131 Naples, Italy; viviana.deluca@ibbr.cnr.it (V.D.L.); vincenzo.carginale@cnr.it (V.C.); 2 Neurofarba Department, Pharmaceutical and Nutraceutical Section, Laboratory of Molecular Modeling Cheminformatics & QSAR, University of Florence, Via Ugo Schiff 6, Sesto Fiorentino, 50019 Florence, Italy; andrea.angeli@unifi.it (A.A.); alessio.nocentini@unifi.it (A.N.); paola.gratteri@unifi.it (P.G.); 3 Department of Chemistry “Ugo Schiff”, University of Florence, Via Della Lastruccia 3-13, Sesto Fiorentino, 50019 Florence, Italydamiano.tanini@unifi.it (D.T.); antonella.capperucci@unifi.it (A.C.)

**Keywords:** COVID-19, SARS-CoV-2, main protease, inhibitor, solubility, hydrolysis, coronavirus

## Abstract

The implementation of innovative approaches is crucial in an ongoing endeavor to mitigate the impact of COVID-19 pandemic. The present study examines the strategic application of the SARS-CoV-2 Main Protease (M^pro^) as a prospective instrument in the repertoire to combat the virus. The cloning, expression, and purification of M^pro^, which plays a critical role in the viral life cycle, through heterologous expression in *Escherichia coli* in a completely soluble form produced an active enzyme. The hydrolysis of a specific substrate peptide comprising a six-amino-acid sequence (TSAVLQ) linked to a p-nitroaniline (pNA) fragment together with the use of a fluorogenic substrate allowed us to determine effective inhibitors incorporating selenium moieties, such as benzoselenoates and carbamoselenoates. The new inhibitors revealed their potential to proficiently inhibit M^pro^ with IC_50_-s in the low micromolar range. Our study contributes to the development of a new class of protease inhibitors targeting M^pro^, ultimately strengthening the antiviral arsenal against COVID-19 and possibly, related coronaviruses.

## 1. Introduction

The COVID-19 pandemic caused by the coronavirus SARS-CoV-2 has reached its third year with alarming numbers [1]. During this period, the virus generated over 6.9 million deaths, of which approximately 190,000 were in Italy. However, the scenario was also accompanied by damage to culture, economy, and social life in general [2,3]. The pandemic has dramatically and negatively changed the way we all live [4]. Perhaps initially, science was caught off guard by the violence of the virus’s attack. However, it later performed “miracles” by producing vaccines against the responsible virus in record time, which was unimaginable before the pandemic [5]. Vaccines have remained critical for protecting the global population. However, the continued emergence of variants, which is exceptionally high in the case of SARS-CoV-2, has not allowed the immunological approach to eradicate the virus [6]. It has only resulted in coexistence with an attenuated form of it. Unfortunately, the concept of coexistence, which has become widespread, is a partial victory, not a final victory, because it accepts the genuine risk of the emergence of more pathogenic viral variants that “pierce” immunity, triggering an exhausting race between new variants and new efficient vaccines [7].

Therefore, it is evident that there is a need to associate the development of new drugs capable of eliminating the virus with vaccine prevention, thus definitively ending the threat posed by this virus [8,9]. At the onset of the pandemic, numerous studies focused on researching ways to treat severely ill COVID-19 patients to save lives and alleviate the burden on hospitals [10]. In mid-2020, scientists discovered that a steroid, dexamethasone, suppresses overstimulated immune responses that can contribute to the final stages of severe illness and reduce deaths among this group [11,12]. These steroids remain the most effective treatment for reducing deaths from COVID-19. Other drugs target the virus more directly, but must be administered by healthcare professionals, which limits their use. The field of selenium-containing drugs is in its nascent stage. However, significant evidence supports the impact of selenium properties on the pharmacological activity, toxicity, and biochemical pathways of organoselenium compounds. In recent years, various structurally diverse organoselenium compounds have emerged, exhibiting promising chemopreventive and antioxidant activities [13,14,15,16,17]. The introduction of selenium into small molecules often brings about additional benefits that are closely related to the modulation of the oxidative stress status of mammalian cells [13,14,15,16,17]. In addition, organoselenium compounds have exhibited both anti-SARS-CoV-2 activity and antioxidant/anti-inflammatory properties, positioning them as potential antiviral and anti-COVID-19 agents. Ebselen, for instance, has demonstrated inhibitory effects on both the SARS-CoV-2 proteases, with an IC_50_ ranging from 0.67 to 2.1 μM [18,19]. This underscores the potential of exploring organoselenium compounds for the development of drugs targeting COVID-19 [20]. Hence, in our study, we sought to address this potential, by synthesizing and investigating novel organoselenium derivatives against the viral recombinant M^pro^ as potential anti-SARS-CoV-2 agents.

**Figure 1:**
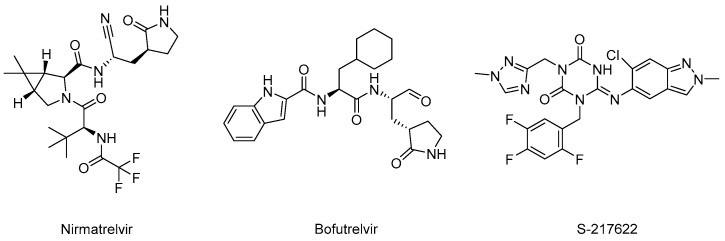
Clinically used M^pro^ inhibitors Nirmatrelvir and Bofutrelvir and S-217622 in clinical trials.

The antiviral drug remdesivir (Veklury), a viral RNA polymerase inhibitor, was administered as an injection and, therefore, until recently, it was only reserved for hospitalized patients with COVID-19 [21,22]. Several companies have developed monoclonal antibodies, which are mass-produced versions of neutralizing antibodies that the immune system synthesizes to bind to SARS-CoV-2 and deactivate it [23,24]. These therapies have offered another avenue for early treatment, and more than 200 monoclonal antibodies are currently in development or awaiting authorization [25,26]. However, they are expensive compared to other treatments, often in short supply, and typically require injection [27]. A recent exception is the long-acting combination of two monoclonal antibodies (mAbs) called Evusheld [28]. This drug can be injected into the muscle and was authorized by the FDA in December to prevent COVID-19 in people at high risk of exposure to SARS-CoV-2 [28]. Over time, attention has shifted towards drugs that can be used outside the hospital environment to treat mild disease, hoping to prevent progression towards a more severe form [29]. Numerous antivirals have been developed to block viral replication by targeting one of its crucial proteins [30]. One such example is molnupiravir, which targets the viral RNA-dependent RNA polymerase [31,32]. Molnupiravir is a prodrug metabolized into an active form, acting as a mutagenic ribonucleoside analog, and inducing mutations in the viral RNA during replication, which can cause errors and prevent the virus from replicating correctly [32]. Other molecules, such as Nirmatrelvir or Bofutrelvir (Figure 1), operate by blocking the main protease (M^pro^) of SARS-CoV-2, which is responsible for cutting viral polyproteins into their final functional forms [33,34]. Neither drug is a panacea, as molnupiravir may cause mutations in human DNA, leading regulatory bodies to discourage its use during pregnancy [31]. Meanwhile, the use of Nirmatrelvir in combination with Ritonavir (known as Paxlovid), although leading to a wide range of pharmacological interactions with commonly used drugs, seems to be the most effective clinical avenue to date [35,36]. Recently, among these new protease inhibitors, the one that has made the most progress is S-217622, which is in an advanced stage of clinical testing [37].

Here, we decided to clone, express, and purify the SARS-CoV-2 M^pro^. Our data showed that SARS-CoV-2 M^pro^ may be expressed successfully in a fully soluble form using the *Escherichia coli* BL21(DE3) strain and with a relatively simple protocol. Affinity chromatography was used for purification of M^pro^. This viral protease will be used in our laboratories to discover new, effective, and low-side-effect small molecules that can inhibit the replication of SARS-CoV-2, contributing to the development of future antiviral strategies and therapeutic interventions.

## 2. Results and Discussion

### 2.1. Cloning, Expression, and Purification

**Figure 2:**
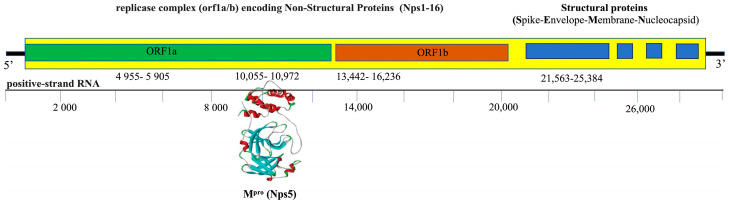
The genome organization of coronaviruses is depicted schematically. The genome comprises positive-strand RNA with nucleotide positions indicated by numbers. The 5′-UTR (represented by a black line on the left) is followed by ORF1a and b (green and brown boxes), respectively, which encode 16 nonstructural proteins (Nsp1–16). Only the specific nonstructural protein, M^pro^ (Nsp5), is indicated in this representation. Additionally, the schematic includes the ORF (blue boxes), encoding four viral structural proteins: S (spike), E (envelope), M (membrane), and N (nucleocapsid). The black line on the right side represents the 3′-UTR.

The viral genome of SARS-CoV-2 is composed of a positive-sense, single-stranded RNA molecule (Figure 2).

It consists of several structural and nonstructural genes that encode proteins necessary for viral replication and infection [38,39]. SARS-CoV-2 M^pro^ is encoded by a specific gene known as ORF1ab, which is located in the open reading frame (ORF) 1ab region of the viral genome (Figure 2). The examination of SARS-CoV-2 M^pro^ has provided opportunities to develop drugs that can effectively combat coronaviruses [38,39,40,41]. M^pro^ is known to operate on at least 11 different points on the large polyprotein 1ab, also known as replicase 1ab, which has a size of approximately 790 kDa and is the largest gene in the viral genome [38]. Most of these sites contain a recognition sequence composed of Leu-Gln, followed by a cleavage site that can be either Ser, Ala, or Gly [42]. One advantage of targeting M^pro^ for inhibition is that its cleavage specificity is unique to coronaviruses and differs from that of human proteases [43]. This specificity arises from the specific amino acid sequence and structural characteristics of the M^pro^ active site [44,45]. As a result, inhibitors designed to target M^pro^ are less likely to interfere with human proteases and, therefore, may be less prone to causing toxicity or adverse effects in human cells [45]. Expressing and purifying the SARS-CoV-2 M^pro^ is crucial for conducting structural and functional research on this protein.

**Figure 3:**
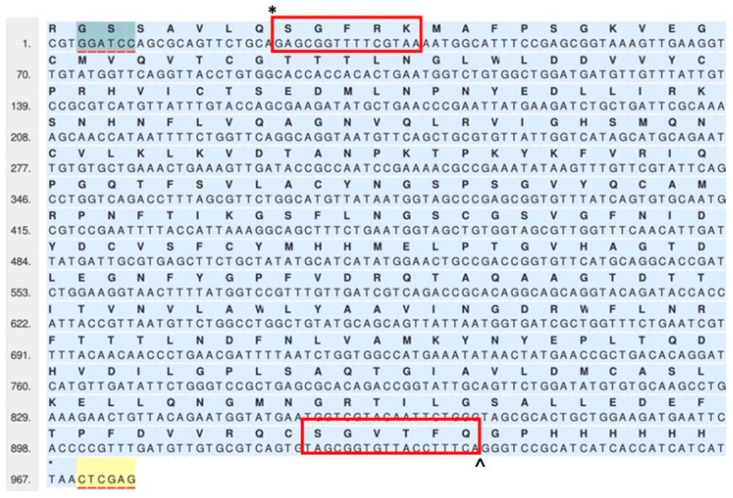
Amino acid and nucleotide sequences of SARS-CoV-2 M^pro^. Legend: amino acid residues are indicated in uppercase bold letters; the nucleotide residues optimized for *E. coli* expression are reported in uppercase letters; the red underlined nucleotide residues indicate the BamHI restriction site (highlighted in gray) and the XhoI restriction site (highlighted in yellow); SAVLQ*SGFRK, M^pro^ cleavage site; SGVTFQ^GPHHHHHH, PreScission cleavage site and the Tag of six histidines; * and ^ are the cleavage sites; the two red boxes indicate the native M^pro^ N- and C-terminals obtained after auto-cleavage and treatment with PreScission protease.

Based on the primary structure of the SARS-CoV-2 M^pro^, a synthetic gene was synthesized to encode the M^pro^ polypeptide chain, which consists of 322 residues (Figure 3).

The gene construct had start and stop codons, and sites placed at appropriate intervals to allow the expression of the native N- and C-terminals of M^pro^. The construct contained the M^pro^ cleavage site SAVLQ*SGFRK (* represents the scissile peptide bond) at the N-terminus. A PreScission cleavage site [46] and a Tag of six histidines (SGVTFQ^GPHHHHHH, the symbol ^ indicates the PreScission cleavage site) were inserted at the C-terminus [47]. In the process of gene expression, the M^pro^ enzyme undergoes auto-cleavage to generate a native N-terminus, whereas, after treatment with PreScission protease, the original C-terminus is developed. The recombinant mature enzyme was a polypeptide chain of 306 amino acid residues, starting with SGFRK and terminating with SGVTFQ (Figure 3, boxed residues).

**Figure 4:**
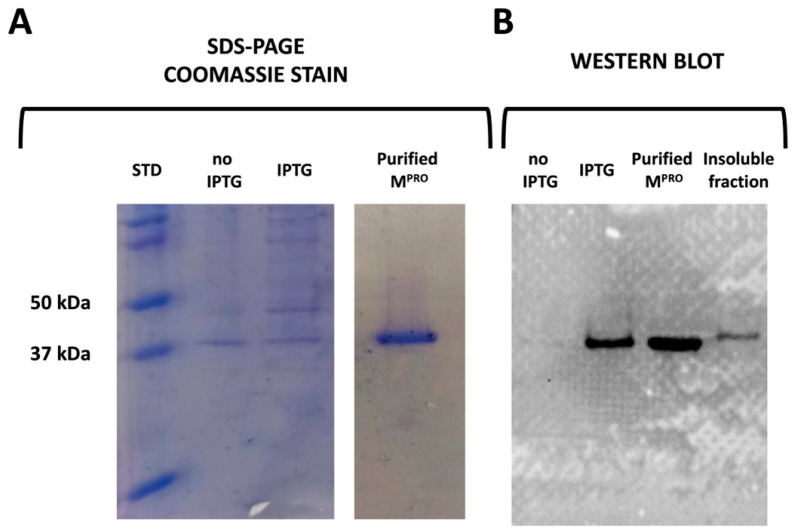
Recombinant M^pro^ protein was analyzed using a combination of SDS-PAGE and Western Blotting. Samples without IPTG induction, samples induced with IPTG, and affinity-purified M^pro^ protein were subjected to SDS-PAGE to assess their protein profiles (Panel **A**). Furthermore, the samples, including the pellet from the sonication step, were subjected to electroblotting and subsequent incubation with an anti-HisTag antibody (Panel **B**). Molecular markers were incorporated as a reference standard to ensure accurate size determination. Legend: Lane STD, molecular markers; Lane no IPTG, cells withdrawn before induction; Lane IPTG, cells withdrawn after adding IPTG and 18 h of incubation at 20 °C; Lane, Purified M^pro^, viral proteinase after HisTrap affinity column; Lane Insoluble fraction, pellet after sonication and centrifugation.

The recombinant SARS-CoV-2 M^pro^ was successfully obtained from soluble fractions of bacterial cell lysates. This was achieved by cultivating bacterial cells containing the SARS-CoV-2 M^pro^/pET-100D Topo plasmid under specific conditions, such as a lower temperature (20 °C) and a mild concentration of IPTG (0.5 mM). The expression and purification of the recombinant M^pro^ were evaluated through SDS-PAGE and Western Blot analysis (Figure 4A,B).

Upon IPTG induction, M^pro^ is expressed at a relatively high level. Notably, most of the expressed M^pro^ was found in the soluble fraction of the bacterial cell lysates, which were purified using a HisTrap affinity column (Figure 4A, right side). The expression of His-tagged M^pro^ was confirmed by Western Blot analysis using an anti-His-tag antibody (Figure 4B). The antibody-detected protein had an apparent molecular weight of approximately 37.0 kDa, whereas its calculated theoretical molecular mass was 34.7 kDa (Figure 4B). Purified His-tagged M^pro^ was treated with PreScission protease at 4 °C overnight to achieve the desired authentic C-termini for the target protein. Subsequently, PreScission-treated M^pro^ was passed against the HisTrap affinity column to eliminate the cleaved His-tagged fragments produced by the PreScission protease. A yield of 4 mg of recombinant M^pro^ (theoretical molecular weight of 33.7 kDa) was obtained from a 1 L culture of bacterial cells.

### 2.2. Determination of the M^pro^ Activity Using the Spectrophotometric Method

**Figure 5:**
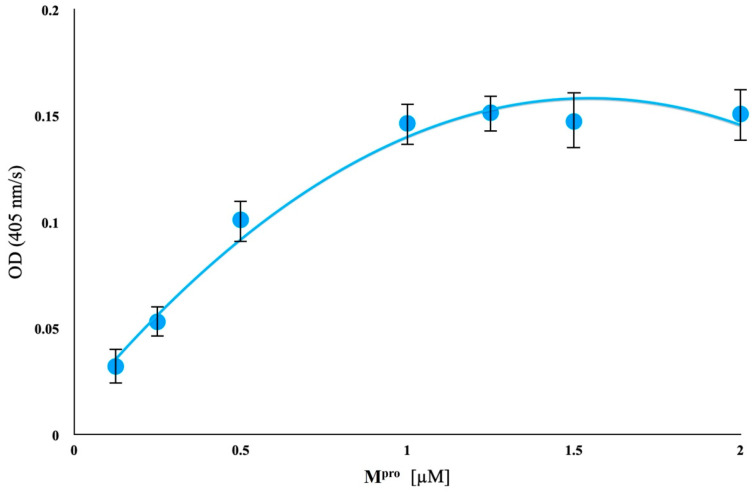
Dependence of M^pro^ activity on enzyme concentration. The enzyme concentrations (from 0.125 µM to 2.0 µM) were incubated with a fixed substrate concentration (500 µM) at a temperature of 22 °C. The protease substrate cleavage was continuously monitored for 40 s. By observing the changes in optical density at 405 nm over time, the graph allowed for the assessment of how varying enzyme concentrations impacted the activity of M^pro^ in catalyzing the cleavage of the substrate. Data represent the mean of three independent experiments.

To evaluate the recombinant M^pro^ enzymatic activity, the hydrolysis of a specific substrate peptide was investigated. The peptide comprises a sequence of six amino acids (TSAVLQ) connected to a p-nitroaniline (pNA) moiety at the amino terminal part [48]. The substrate exhibits a remarkable susceptibility to precise cleavage by M^pro^ at a designated cleavage site known as Gln-pNA. The proteolytic activity of M^pro^ was assayed continuously by monitoring the cleavage of the TSAVLQ-pNA. The enzymatic action executed by M^pro^ leads to the liberation of pNA, which elicits a noticeable increase in absorbance at the wavelength of 405 nm. By monitoring the hydrolysis of this substrate peptide, we gain valuable insights into the enzymatic proficiency of M^pro^. Figure 5 shows the dependence of enzyme activity, expressed as optical density 450 nm/s, as a function of the enzyme concentration, which was in the range of 0.125–2 µM.

Based on the insights provided by Figure 5, it is evident that the purified recombinant enzyme exhibits good activity, with the reaction rate influenced by the enzyme concentration. However, additional increments in enzyme concentration (M^pro^ > 1.2 µM) cease to impact the reaction rate. This intriguing observation can be attributed to the gradual imposition of a limiting factor due to the substrate’s availability (500 µM in the assay). Moreover, we also investigated the M^pro^ activity of the recombinant protein with the native N-terminus (SGFRK) but having the PreScission cleavage site and the tag of six histidines at the C-terminal (SGVTFQ^GPHHHHHH). The M^pro^ with only the native N-terminus resulted in behavior similar to that of the protease with the native N- and C-terminals in the polypeptide chains. This result was expected since the native N-terminal, not the M^pro^ C-terminal, is crucial for the protease dimerization process [49]. More specifically, the interaction of the Ser in the first position of one monomer with Glu166 present in the adjacent monomer is crucial for the catalytic activity of the viral protease [49].

### 2.3. Determination of the M^pro^ Half Maximal Inhibitory Concentrations (IC_50_)

Initially, in our analysis we also employed a colorimetric method to assess M^pro^ activity and inhibition. However, recognizing the limitations of colorimetric assays, we sought to enhance the accuracy of our method. To this end, we transitioned to a more sensitive approach using a fluorogenic substrate for enzyme inhibition analysis. The fluorogenic substrate was preferred over the colorimetric method due to its higher sensitivity, ability to detect subtle changes in fluorescence intensity, and broader dynamic range for IC_50_ determination. Moreover, the fluorogenic substrate enabled a more refined evaluation of compound efficacy, particularly for detecting lower concentrations. By mitigating background noise and interference, the fluorogenic substrate also improved the reliability and accuracy of the experimental measurements. A plethora of inhibitors targeting SARS-CoV-2 M^pro^ have been evaluated, and few have demonstrated low IC_50_ values [50,51,52]. In the literature it has been reported that Nirmatrelvir and Bofutrelvir (Figure 1) exhibit IC_50_ values of less than 1 μM when tested against SARS-CoV-2 M^pro^ [50,51,52]. Nirmatrelvir, a drug marketed under the trade name Paxlovid (in combination with Ritonavir), and Bofutrelvir, are two orally administered antiviral therapeutics specifically developed by Pfizer for the management of COVID-19 [53,54,55]. These two inhibitors were demonstrated to disrupt a critical stage of the viral life cycle, potentially reducing disease progression and alleviating the severity of COVID-19 symptoms. It is essential to note that ongoing clinical investigations and regulatory evaluations continue to assess the efficacy, safety profile, and optimal administration protocols for these and other M^pro^ inhibitors [53,54,55]. These investigations are crucial for establishing comprehensive therapeutic guidelines and refining treatment strategies to effectively combat COVID-19.

**Figure 6:**
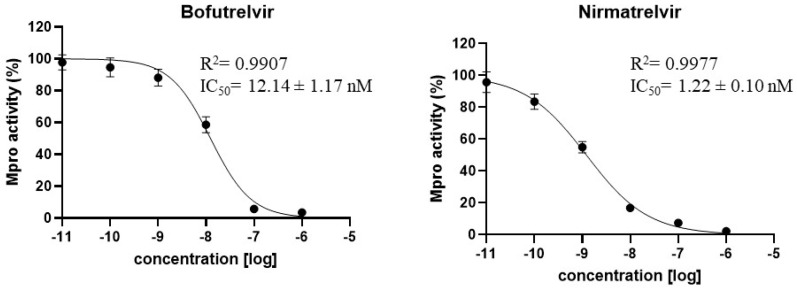
Dose response of M^pro^ activity with Bofutrelvir and Nirmatrelvir. Data represent the mean ± SD (n = 3).

In our study, we determined the IC_50_ values of Nirmatrelvir and Bofutrelvir standard M^pro^ inhibitors, which were used as a positive control for the IC_50_ test. The in vitro experiments demonstrated that Nirmatrelvir and Bofutrelvir had IC_50_ values of 1.22 and 12.14 nM, respectively. These findings are consistent with those previously reported in the literature (see Figure 6).

Selenium-containing inhibitors **2** and **3** were synthesized by exploiting the exquisite nucleophilic character of selenols, which were demonstrated to selectively react with a wide variety of electrophilic partners [56,57,58,59]. Selenols **1** were prepared following reported procedures via reductive cleavage of the corresponding diselenides [60] or through the ring-opening reaction of the corresponding epoxides with (Me_3_Si)_2_Se in the presence of TBAF [59]. The Spectrum of compounds can be seen in the Supplementary Materials.

**Scheme 1:**
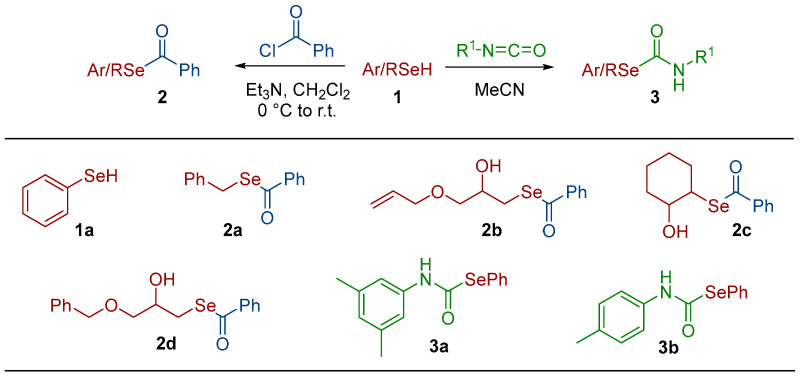
Structure of selenium-containing molecules studied in this work and synthesis of selenolesters **2a**–**d** and selenocarbamates **3a,b**.

Selenolesters **2a**–**d** were synthesized upon reaction of benzyl- or alkyl-substituted selenols with benzoyl chloride in the presence of triethylamine (Scheme 1, left). Similarly, selenocarbamates **3a**,**b** were obtained via the addition reaction of benzeneselenol **1a** to suitable isocyanates (Scheme 1, right) [61,62].

**Scheme 2:**
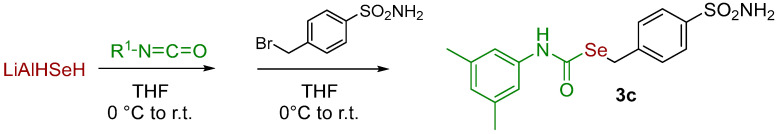
Synthesis of selenocarbamate **3c**.

On the other hand, the synthesis of selenocarbamate **3c** followed a different synthetic pathway involving the use of the selenating reagent LiAlHSeH [62] with isocyanate and reacted with the appropriate benzoyl bromide, as outlined in Scheme 2.

**Figure 7:**
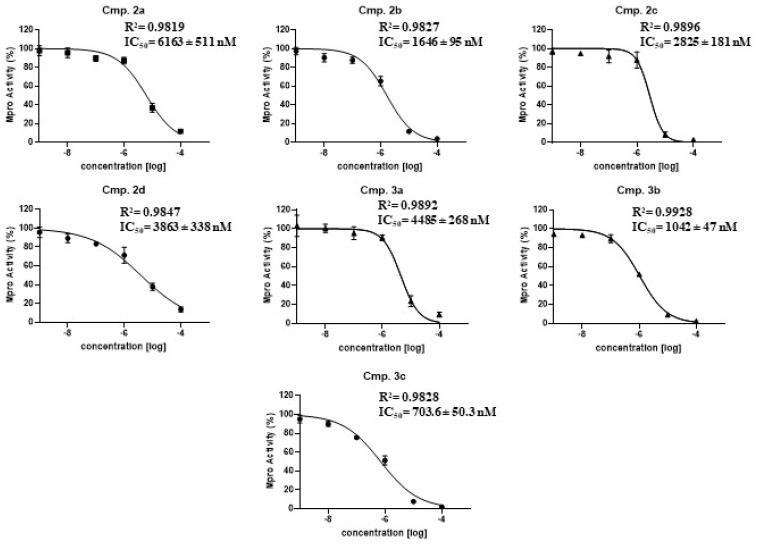
Dose response of M^pro^ activity with organoselenium derivatives **2a**–**d** and **3a**–**c**. Data represent the mean ± SD (*n* = 3).

A fluorogenic assay was conducted to assess the inhibition levels against the M^pro^ enzyme of selenoesters **2a**–**d** and selenocarbamate **3a**–**c** derivatives. In Figure 7, inhibitory curves against M^pro^ and the corresponding IC_50_ values for each compound are presented. The data unequivocally illustrate that these compounds exhibit substantial M^pro^ inhibitory activity, with IC_50_ values in the micromolar range for selenoesters. Notably, derivatives with a hydroxyl group demonstrated better activity than compound **2a** without it.

Conversely, selenocarbamates **3a**–**c** exhibited superior efficacy in inhibiting the M^pro^ enzyme, reaching sub-micromolar levels of inhibition (703.6 nM) for compound **3c**. These findings indicate a clear on-target interaction of these compounds with M^pro^, showcasing significant inhibitory potential against SARS-CoV-2 and suggesting their potential for development as treatments for COVID-19 patients.

### 2.4. Computational Study

**Scheme 3:**
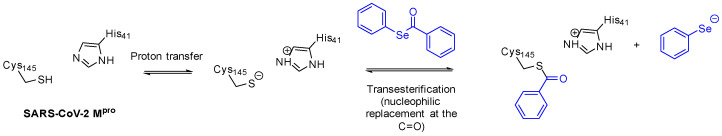
Proposed inhibition mechanism with selenoesters of SARS-CoV-2 M^pro^ by covalent modification of Cys145.

It is noteworthy that computational protein design methods have played a pivotal role in creating novel molecules targeting specific regions of the SARS-CoV-2 virus. Sophisticated computational protein design techniques have been instrumental in generating miniproteins with a high binding affinity for the viral spike protein—an essential element facilitating viral entry into host cells [63]. Additionally, a stapled peptide has been designed, demonstrating both high affinity and specificity for the receptor-binding domain (RBD) of the SARS-CoV-2 spike protein [64]. Considering this, the M^pro^ inhibition mechanism of selenoesters **2a**–**2d** and selenocarbamates **3a**–**3c** was simulated by a covalent docking analysis selecting Cys145 as the bond forming residue (Scheme 3).

**Figure 8:**
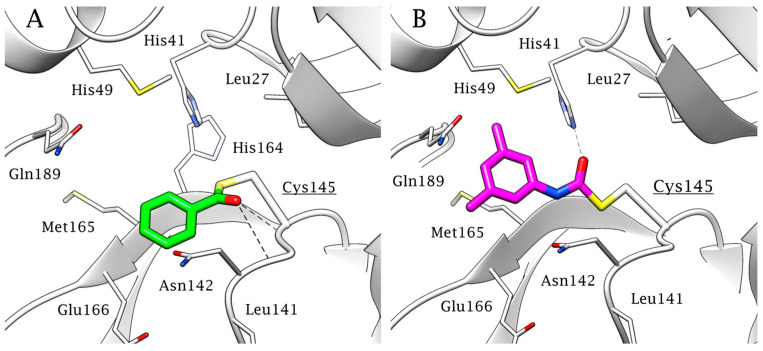
Mpro inhibition mechanism predicted by covalent docking for (**A**) selenoesters **2a**–**2d** and (**B**) selenocarbammates **3a**–**3c**.

A series of thioester inhibitors of SARS-CoV-2 M^pro^ has been described previously and X-ray structures showed a covalent thioester bond with the catalytic Cys145 residue of the protease [65]. Modifying the force field (OPLS4) allowed us here to simulate similar selenium derivatives. The results indicate that a nucleophilic attack of the cysteine thiol to the selenoester or selenocarbamate carbonyl group occurs with the related selenides acting as the leaving groups and leads to the formation of the covalently bound thioester or thiocarbammate adduct (Figure 8). The thioester carbonyl group is predicted to engage H-bonds with the side chain NH moieties of Gly143 and Cys145 (Figure 8A). The benzene ring accommodates in the S2 pocket of the M^pro^ active site forming VdW contacts with residues nearby. Instead, the thiocarbammate carbonyl group forms a H-bond with the His41 imidazole ring (Figure 8B). The methyl substituted phenyl ring is located in the same enzyme subpocket but shifted towards Gln189 and Met49 due to the greater length of the linker, confirming thus the hypothesized binding proposed in Scheme 3.

## 3. Materials and Methods

### 3.1. Construction of M^pro^ Expression Vector

The M^pro^ gene for SARS-CoV-2, which covers ORF1ab polyprotein residues 3264–3569 and has a GenBank code of MN908947.3, was designed in our laboratories and produced by GeneArt (Life Technologies, Carlsbad, CA, USA), a company specialized in gene synthesis. The gene was synthesized using *Escherichia coli* codon usage. The vector pET100D-Topo was used to produce the pET100/M^pro^ vector, which overexpressed the fusion recombinant viral protease with a Tag of six histidines at the C-terminus of the polypeptide chain. An intricate design was employed for gene construction by incorporating strategic nucleotide sites. At the beginning of the start codon, the nucleotides encoding the amino acids of the M^pro^ cleavage site SAVLQ*SGFRK were added (* is the M^pro^ autocleavage). In contrast, the nucleotides encoding amino acids for the PreScission cleavage site and six histidines (SGVTFQ^GPHHHHH) were added before the stop codon (^ is the PreScission cleavage site). This meticulous arrangement ensured the accurate expression of the native N- and C-terminals of the native M^pro^ through autocleavage and PreScission protease, respectively (see Results and Discussion for more details).

### 3.2. M^pro^ Expression and Purification

To overexpress His-Tag M^pro^, competent *E. coli* BL21 (DE3) cells were transformed with the constructs described above. They were grown at 37.0 °C and induced with 0.5 mM isopropyl-thio-b-D-galactoside (IPTG) at an OD600 of 0.6–0.7 nm. After additional growth for 18 h at 20 °C, the cells were harvested by centrifugation and washed three times with PBS 1X. Aliquots of cells were resuspended in 20 mM Tris/HCl, 250 mM NaCl, 2 mM *β*-Mercaptoethanol, 0.2% Triton, pH 8.5, and disrupted by sonication. The bacterial lysate was centrifuged and purified using a nickel-affinity column (His-Trap FF). HisTrap column (1.0 mL) was equilibrated with 20 mL equilibration buffer (20 mM Tris/HCl, 250 mM NaCl, 2 mM *β*-Mercaptoethanol, 0.2% Triton, pH 8.5) at 1 mL/min. The supernatant from the cellular lysate was loaded onto the column at 1.0 mL/min, connected with AKTA Prime. The recombinant His-Tag M^pro^ was eluted from the column with a flow of 0.5 mL/min and the elution buffer composed of 20 mM Tris/HCl, 30 mM NaCl, 2 mM *β*-Mercaptoethanol, 150 mM imidazole, pH 8.5. The fractions containing target protein were pooled and mixed with PreScission protease and dialyzed into 20 mM Tris/HCl, 150 mM NaCl, 2 mM *β*-Mercaptoethanol, pH 8.5 at 4 °C overnight, resulting in the target protein with authentic N- and C-termini. PreScission-treated M^pro^ was applied again to the His-Trap FF nickel columns to remove the PreScission protease, His-tag, and protein with an uncleaved His-tag. The His-tag-free M^pro^ in the flow through was dialyzed into 50 mM HEPES, 150 mM NaCl, 1 mM DTT, 1 mM EDTA, and 10% glycerol, pH 7.5 at 4 °C overnight.

### 3.3. SDS-Page and Western Blot

A 12% Sodium Dodecyl Sulfate-polyacrylamide gel electrophoresis (SDS-PAGE), following the method described by Laemmli, was used to load and separate the recovered M^pro^ at various steps of the purification process. The gel was electrophoresed at 150 V until the dye front completely migrated off the gel and subsequently stained with Coomassie Brilliant Blue-R for visualization. For the subsequent Western Blot analysis, the overexpressed cytoplasmic M^pro^ was subjected to electrophoretic transfer onto a PVDF membrane using a transfer buffer composed of 25 mM Tris, 192 mM glycine, and 20% methanol using a Trans-Plot SD Cell (Bio-Rad, Hercules, CA, USA). His-tag Western Blot was carried out using the Pierce Fast Western Blot Kit (Thermo Scientific, Waltham, MA, USA). The blotted membrane was immersed in Fast Western 1 Wash Buffer to eliminate any residual transfer buffer. The working dilution of the primary antibody was then applied to the blot and incubated for 30.0 min at room temperature (RT) with gentle agitation. Following this, the membrane was removed from the primary antibody solution and incubated for 10.0 min with Fast Western Optimized HRP Reagent Working Dilution. The membrane was washed twice using approximately 20 mL of Fast Western 1 Wash Buffer. Finally, the membrane was incubated with the Detection Reagent Working Solution for 3.0 min at RT. The chemiluminescent signals were captured using the Invitrogen iBright CL1500 Imaging System, enabling data acquisition and analysis.

### 3.4. Enzymatic Protease Assay

The enzymatic activity of M^pro^ was evaluated using a colorimetric assay that measured the peptide cleavage of the peptide substrate TSAVLQ-para-nitroanilide (TQ6-pNA, Merck, Rahway, NJ, USA) [48]. This substrate undergoes cleavage at the Gln-pNA bond, resulting in the release of free pNA and a visible change in the solution color to yellow. The absorbance was continuously monitored at 405 nm using a Varian Cary 50 UV-Vis Spectrophotometer (Palo Alto, CA, USA). Protease activity assay was performed at 22 °C in 20 mM phosphate buffer (pH 7.6). The substrate stock solution was prepared at a concentration of 1 mM and the working concentration used in the assay was 500 µM. The assay was assembled in a quartz cuvette (0.5 mL) containing the enzyme in working protein solutions ranging from 0.125–2 µM.

For the determination of half-maximal inhibitory concentrations (IC_50_) of inhibitors against M^pro^, an AFC-Peptide substrate (SAE0180, Merck) was used for a fluorescence-based cleavage assay [66]. The assay was performed in half area 96-well, black, flat-bottomed microtiter plates (Corstar, Corning, Glendale, CA, USA) with a final volume of 125 μL. M^pro^ (final concentrations of 5–50 nM) was pre-incubated for 1 h at 25 °C with compounds at different concentrations in the assay buffer (PBS, pH 7.5). The substrate was then added at a final concentration of 12 μM to the reaction mixture and the reaction was incubated for 1 h at 25 °C. The readings for the different concentrations of the inhibitor compounds incubated with the substrate without M^Pro^ were measured as a blank. The fluorescence signals (excitation/emission, 400 nm/505 nm) of released AFC were measured using a Spark multimode plate reader (Tecan, Glendale, CA, USA). The results were plotted as dose inhibition curves using nonlinear regression with a variable slope to determine the IC_50_ values of inhibitor compounds using GraphPad Prism 9.0.

### 3.5. Chemistry

#### 3.5.1. General

All commercial materials were purchased from Merck—Sigma-Aldrich and used as received, without further purification. Solvents were dried using a solvent purification system (Pure-Solv™). Flash column chromatography purifications were performed with Silica gel 60 (230–400 mesh). Thin layer chromatography was performed with TLC plates Silica gel 60 F254, which was visualized under UV light, or by staining with an ethanolic acid solution of *p*-anisaldehyde followed by heating. Mass spectra were recorded by Electrospray Ionization (ESI). ^1^H and ^13^C NMR spectra were recorded in CDCl_3_ or DMSO-*d*_6_ using Varian Mercury 400 and Bruker 400 Ultrashield spectrometers, operating at 400 MHz for ^1^H, 100 MHz for ^13^C, and 76 MHz for ^77^Se. ^1^H NMR signals were referenced to nondeuterated residual solvent signals (7.26 ppm for CDCl3 and 2.49 for DMSO-*d*_6_). ^13^C NMR was referenced to CDCl_3_ or DMSO-*d*_6_ signals (77.0 ppm or 39.7 ppm, respectively). (PhSe)_2_ was used as an external reference for ^77^Se NMR (*δ* = 461 ppm). Chemical shifts (*δ*) are given in parts per million (ppm) and coupling constants (*J*) are given in Hertz (Hz), rounded to the nearest 0.1 Hz. The 1H NMR data are reported as follows: chemical shift, integration, multiplicity (s = singlet, d = doublet, t = triplet, ap d = apparent doublet, m = multiplet, dd = doublet of doublet, bs = broad singlet, bd = broad doublet, etc.), coupling constant (*J*) or line separation (ls), and assignment. Spectroscopic data of compounds **2b, 2d**, **3a**, and **3b** matched those reported in the literature.

#### 3.5.2. General Procedure for the Synthesis of Selenolesters (**2**)

A solution of selenol **1** (1.0 mmol) in dry CH_2_Cl_2_ (3 mL) was cooled at 0 °C under inert atmosphere (N_2_) and treated with Et_3_N (1.2 mmol). The mixture was stirred for 5 min and then a solution of acyl chloride (1.2 mmol) in dry CH_2_Cl_2_ (2 mL) was slowly added. The reaction was allowed to warm to room temperature and stirred for an additional 2 h. Afterwards, a saturated solution of *aq*. NH_4_Cl was added and the organic phase was extracted with Et_2_O (3 × 15 mL), washed with brine (2 × 10 mL) and dried over Na_2_SO_4_. The solvent was removed under vacuum and the crude material was purified by flash column chromatography (silica gel) to afford pure selenolesters **2**.

#### 3.5.3. Synthesis of Se-Phenethyl Benzoselenoate (**2a**)

Following the general procedure, phenylmethaneselenol (86 mg, 0.5 mmol) and benzoyl chloride, after purification by column chromatography (petroleum ether/Et_2_O 15:1), gave **2a** as a yellowish oil (74 mg, 51%). **^1^H NMR** (400 MHz, CDCl_3_), *δ* (ppm): 4.35 (2H, s), 7.22 (1H, t, *J* = 7.2 Hz), 7.30 (2H, ap t, ls = 7.5 Hz), 7.38 (2H, ap d, ls = 7.2 Hz), 7.45 (2H, ap t, ls = 7.7 Hz), 7.59 (1H, ap t, ls = 7.4 Hz), 7.90 (2H, ap d, ls = 7.4 Hz). **^13^C NMR** (100 MHz, CDCl_3_), *δ* (ppm): 29.0, 127.0, 127.2, 128.6, 128.8, 129.0, 133.7, 138.8, 139.0, 194.5. **MS** (ESI, positive) *m/z*: 291.0 [M + H]^+^.

#### 3.5.4. Synthesis of Se-(2-Hydroxycyclohexyl) Benzoselenoate (**2c**)

Following the general procedure, 2-hydroselenocyclohexan-1-ol (134 mg, 0.75 mmol) and benzoyl chloride, after purification by column chromatography (petroleum ether/EtOAc 5:1), gave **2c** as a yellowish oil (155 mg, 73%). **^1^H NMR** (200 MHz, CDCl_3_) *δ* (ppm): 1.31–1.47 (3H, m), 1.68–1.72 (2H, m), 1.80–1.83 (1H, m), 2.16–2.26 (2H, m), 2.52 (1H, bs), 3.58–3.65 (2H, m), 7.42–7.46 (2H, m), 7.56–7.60 (1H, m), 7.88–7.91 (2H, m). **^13^C NMR** (50 MHz, CDCl_3_) δ (ppm): 24.2, 26.7, 32.9, 35.3, 50.8, 73.4, 127.4, 128.8, 133.8, 139.0, 195.7. **MS** (ESI, positive) *m/z*: 285.0 [M + H]^+^.

#### 3.5.5. Synthesis of Se-(4-Sulfamoylbenzyl) (3,5-Dimethylphenyl) Carbamoselenoate (**3c**)

Elemental selenium (1 eq.) was added in THF at 0 °C and subsequently added LiAlH_4_ (1eq.). The reaction mixture was stirred at 0 °C for 30 min, 1-isocyanato-3,5-dimethylbenzene (1 eq.) was added, and the mixture was then stirred for 1 h at 0 °C. Subsequently, 4-(bromomethyl)benzenesulfonamide (1 eq.) was added, the mixture was then stirred for 2 h, quenched with H_2_O, and extracted with EtOAc. The crude reaction was purified by flash chromatography (EtOAc/Hex 7:3) to afford a yellow solid. Yield 57%. **^1^H NMR** (400 MHz, DMSO-*d*_6_ δ(ppm): 8.48 (1H, s), 7.80 (2H, d, *J* = 8.33 Hz), 7.44 (2H, d, *J* = 8.35 Hz), 7.37 (2H, s), 7.10 (2H, s), 6.64 (1H, s), 4.07 (2H, s), 2.26 (6H, s); **^13^C NMR** (100 MHz, DMSO-*d*_6_) δ(ppm): 153.3, 144.2, 143.5, 140.5, 138.6, 130.2, 126.6, 124.25, 116.8, 31.5, 22.0; **^77^Se NMR** (76 MHz, DMSO-*d*_6_) δ(ppm): 406.1; MS (ESI positive) *m/z*: 399.0 [M + H]^+^.

### 3.6. Computational Study

The crystal structure of SARS-CoV-2 MPro (PDB 7CB7) [67] was retrieved from the Protein Data Bank and prepared using the Protein Preparation module implemented in Maestro Schrödinger suite [68], assigning bond orders, adding hydrogens, deleting water molecules, and optimizing H-bonding networks. Energy minimization with a Root Means Square Deviation (RMSD) value of 0.30 was applied using an Optimized Potential for Liquid Simulation (OPLS4) force field. The 3D ligand structures were prepared by Maestro (version 2023-4) and evaluated for their ionization states at pH 7.4 ± 0.5 with Epik, version 2023-4. The OPLS4 force field was modified according to Schrödinger to enable the treatment of selenium derivatives in the docking procedure. The grid for the covalent docking was generated with the center located on the centroid of the cocrystallized ligand and Cys145 was selected as the reacting residue. Covalent docking was performed with Glide Covalent Docking. The best pose for type of compound, evaluated in terms of score, hydrogen bond interactions and hydrophobic contacts, was refined with Prime adopting a VSGB solvation model. Figures were generated with ChimeraX (version 1.7).

## 4. Conclusions

The study here reported advances our knowledge of viral biochemistry, and may lead to the development of new antivirals belonging to the class of cysteine protease inhibitors. We report conditions for the cloning and purification of M^pro^ with significant yields, which produced highly catalytically active SARS-CoV-2 enzyme. In our study, we successfully synthesized novel selenoester and selenocarbamate derivatives, and evaluated their potential as M^pro^ inhibitors. The screening revealed that all synthesized compounds exhibited inhibition activity against the target enzyme, whereas the selenocarbamate **3c** has emerged as an effective inhibitor, demonstrating an IC_50_ value in the sub-micromolar range at 703.6 nM. These findings underscore the promise of organoselenium derivatives as effective M^pro^ inhibitors, suggesting their potential significance in the development of antiviral therapeutics. Similar to other M^pro^ inhibitors reported so far [69,70], the new compounds investigated here may react with the SH moiety of the cysteine from the catalytic triad of the enzyme, inactivating it. Thus, a possible inhibition mechanism relies on acyl- or carbamoyl-transfer from the selenium atom to the catalytic cysteine of the enzyme. The good leaving group ability of the formed selenolate anions reasonably moves the equilibrium towards the formation of the enzyme-derived thiolesters or thiocarbamate. Such a reactivity has not yet been reported for cysteine proteases from coronaviruses, and thus selenobenzoates and selenocarbamates constitute new classes of M^pro^ inhibitors [71,72]. The success of our synthesized compounds in inhibiting M^pro^ activity contributes valuable insights to the ongoing efforts in identifying and designing compounds for combating viral infections, particularly targeting SARS-CoV-2. Further exploration of the structure–activity relationships and mechanistic studies will enhance our understanding and pave the way for the development of more potent and selective inhibitors with clinical relevance. This study marks a significant stride towards advancing the arsenal of antiviral agents and underscores the potential of organoselenium derivatives in the pursuit of effective treatments against viral infections.

## Data Availability

Data are available directly from the authors at request.

## Supplementary Materials

The following supporting information can be downloaded at: https://www.mdpi.com/article/10.3390/ijms25020971/s1.
