# Supplementary material for: Leveraging SARS-CoV-2 Main Protease (Mpro) for COVID-19 Mitigation with Selenium-Based Inhibitors
Source: Int J Mol Sci. 2024 Jan 12;25(2):971. doi: 10.3390/ijms25020971 (PMC10815619; doi:10.3390/ijms25020971)
Supplement: Supplementary file 1 [file ijms-25-00971-s001.zip › ijms-2788015-Supplementary Material.pdf]

International Journal of *Molecular Sciences*

Supplementary Materials

Leveraging SARS-CoV-2 Main Protease (M^{pro}) for COVID-19 Mitigation with Selenium-Based Inhibitors

Viviana De Luca [1,†], Andrea Angeli [2,†], Alessio Nocentini [2], Paola Gratteri [2], Silvia Pratesi [3], Damiano Tanini [3], Vincenzo Carginale [1], Antonella Capperucci [3], Claudiu T. Supuran [2,*] and Clemente Capasso [1,*]

1 Department of Biology, Agriculture and Food Sciences, National Research Council (CNR), Institute of Biosciences and Bioresources, 80131 Naples, Italy; viviana.deluca@ibbr.cnr.it (V.D.L.); vincenzo.carginale@cnr.it (V.C.)
2 Neurofarba Department, Pharmaceutical and Nutraceutical Section, Laboratory of Molecular Modeling Cheminformatics & QSAR, University of Florence, Via Ugo Schiff 6, Sesto Fiorentino, 50019 Florence, Italy; andrea.angeli@unifi.it (A.A.); alessio.nocentini@unifi.it (A.N.); paola.gratteri@unifi.it (P.G.)
3 Department of Chemistry "Ugo Schiff", University of Florence, Via Della Lastruccia 3-13, Sesto Fiorentino, 50019 Florence, Italy; silvia.pratesi1@stud.unifi.it (S.P.); damiano.tanini@unifi.it (D.T.); antonella.capperucci@unifi.it (A.C.)
* Correspondence: claudiu.supuran@unifi.it (C.T.S.); clemente.capasso@ibbr.cnr.it (C.C.)

Citation: De Luca, V.; Angeli, A.; Nocentini, A.; Gratteri, P.; Pratesi, S.; Tanini, D.; Carginale, V.; Capperucci, A.; Supuran, C.T.; Capasso, C. Leveraging SARS-CoV-2 Main Protease (M^{pro}) for COVID-19 Mitigation with Selenium-Based Inhibitors. *Int. J. Mol. Sci.* **2024**, *25*, 971. https://doi.org/10.3390/ijms25020971

Academic Editor: Barbara Ruaro

Received: 7 December 2023
Revised: 5 January 2024
Accepted: 9 January 2024
Published: 12 January 2024

Copyright: © 2024 by the authors. Licensee MDPI, Basel, Switzerland. This article is an open access article distributed under the terms and conditions of the Creative Commons Attribution (CC BY) license (https://creativecommons.org/licenses/by/4.0/).

Index

^{1}H, ^{13}C ^{77}Se Spectra of compounds **1a**, **2a-c** and **3a-c** *S2–S8*

Copy of NMR Spectra of synthesised compounds

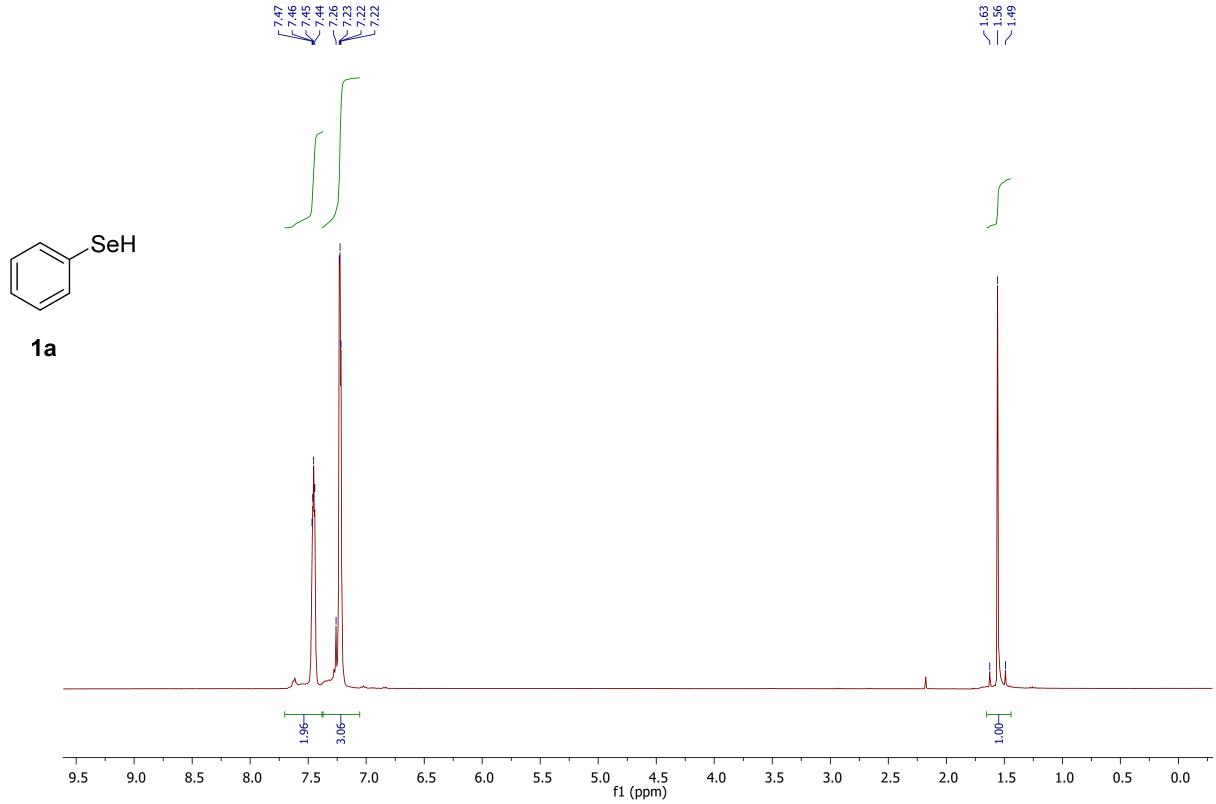

Figure S1. ^{1}H NMR Spectrum of compound **1a** ($CDCl_3$, 400 MHz).

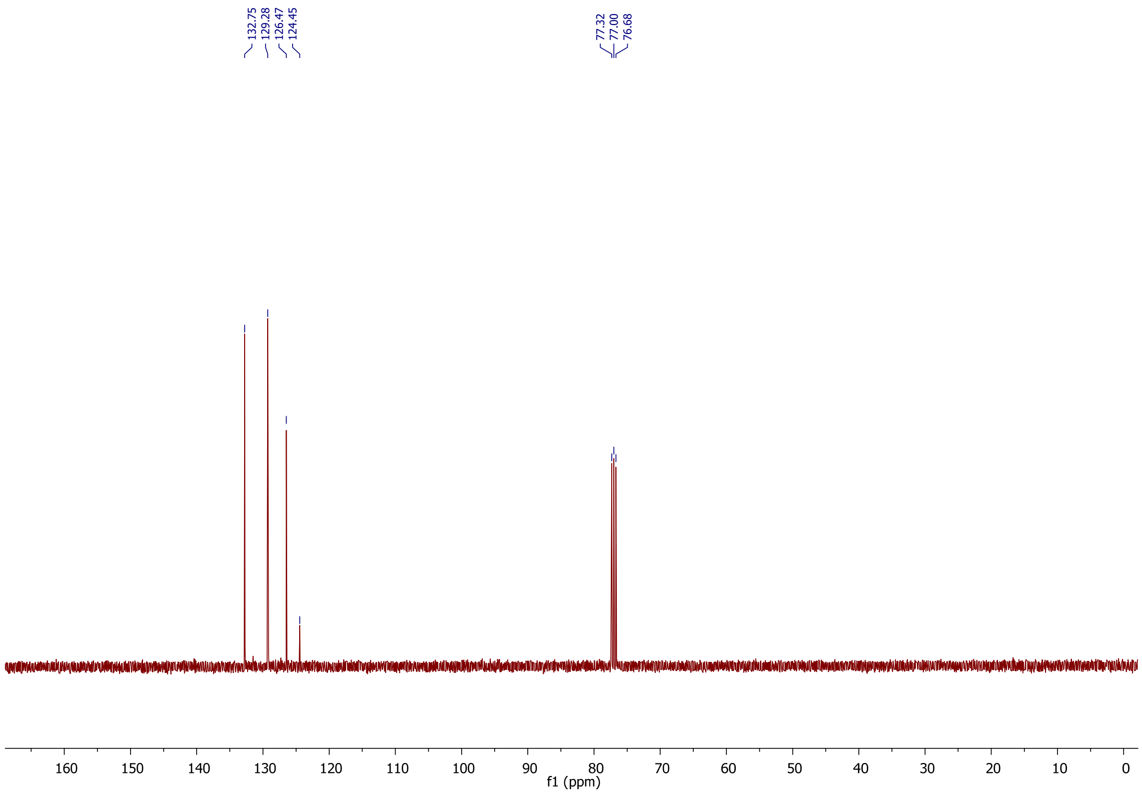

Figure S2. ^{13}C NMR Spectrum of compound **1a** ($CDCl_3$, 100 MHz).

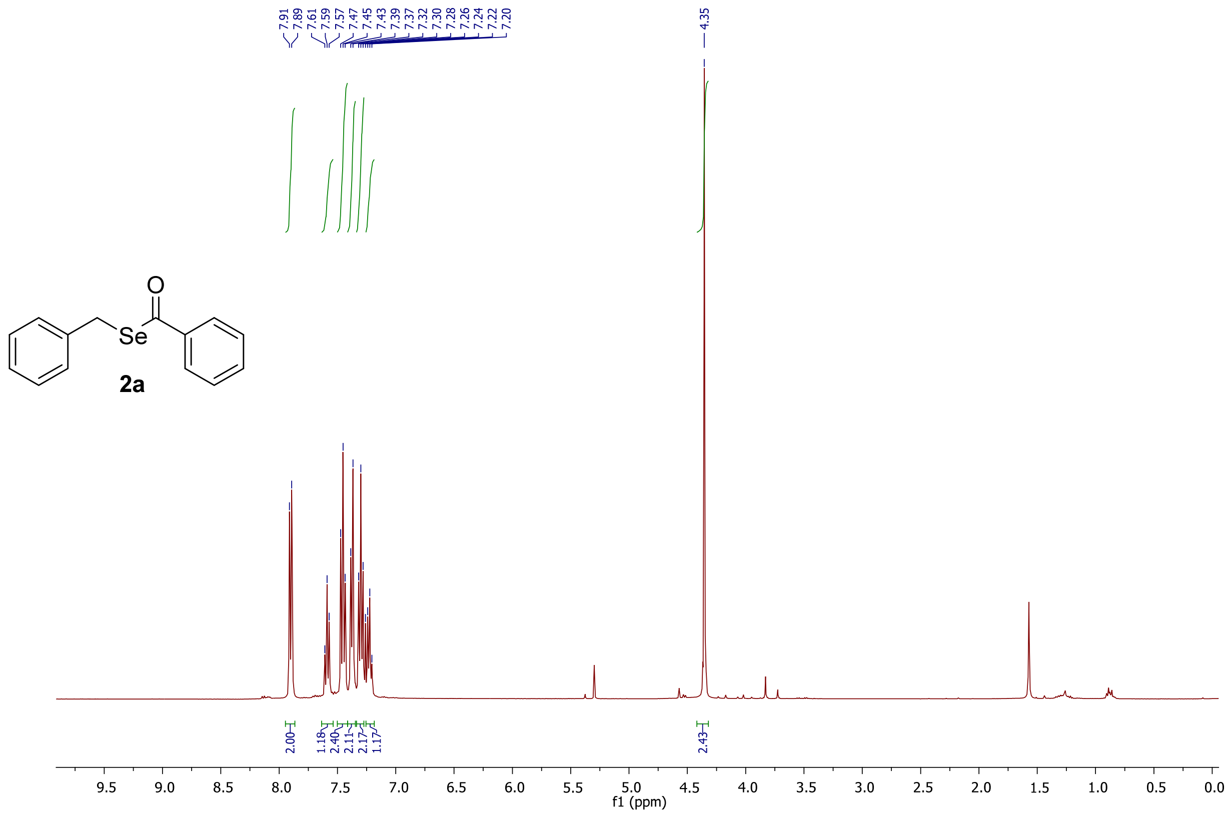

Figure S3. ^{1}H NMR Spectrum of compound **2a** ($CDCl_3$, 400 MHz).

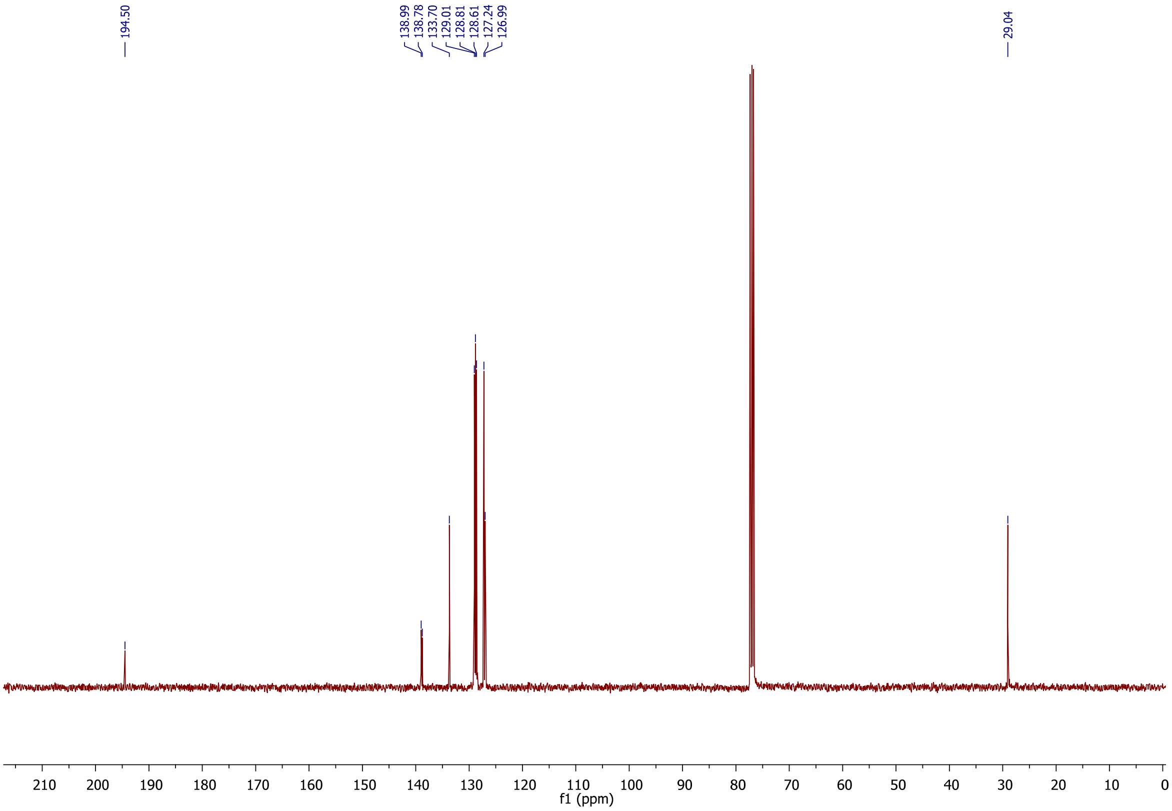

Figure S4. ^{13}C NMR Spectrum of compound **2a** ($CDCl_3$, 100 MHz).

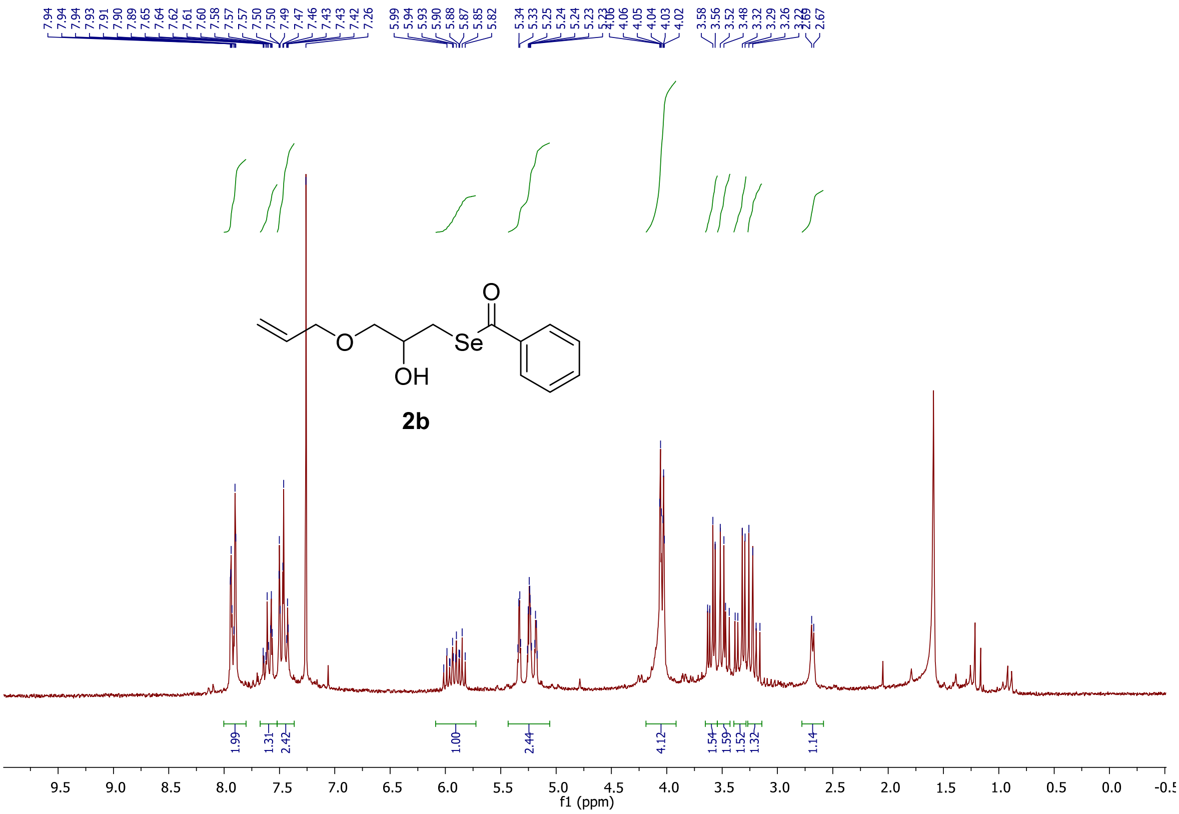

Figure S5. ^{1}H NMR Spectrum of compound **2b** ($CDCl_3$, 200 MHz).

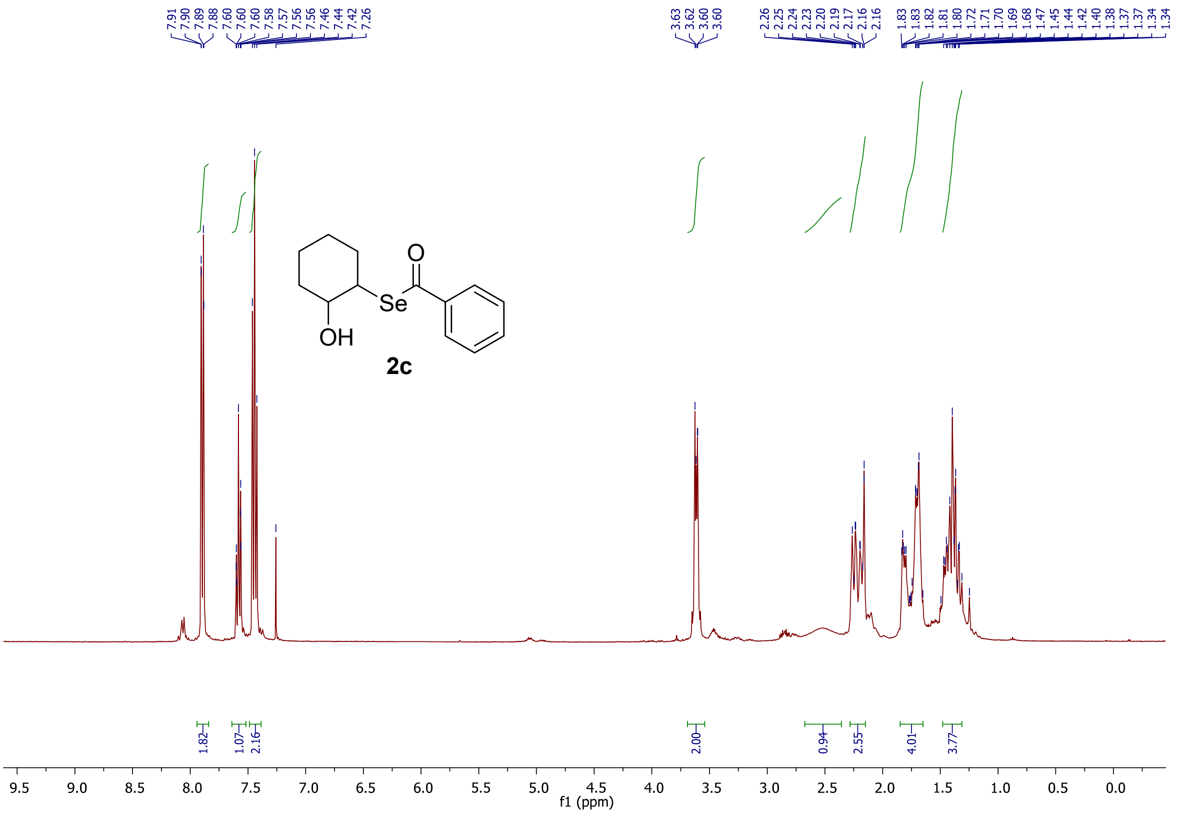

Figure S6. ^{1}H NMR Spectrum of compound **2c** ($CDCl_3$, 400 MHz).

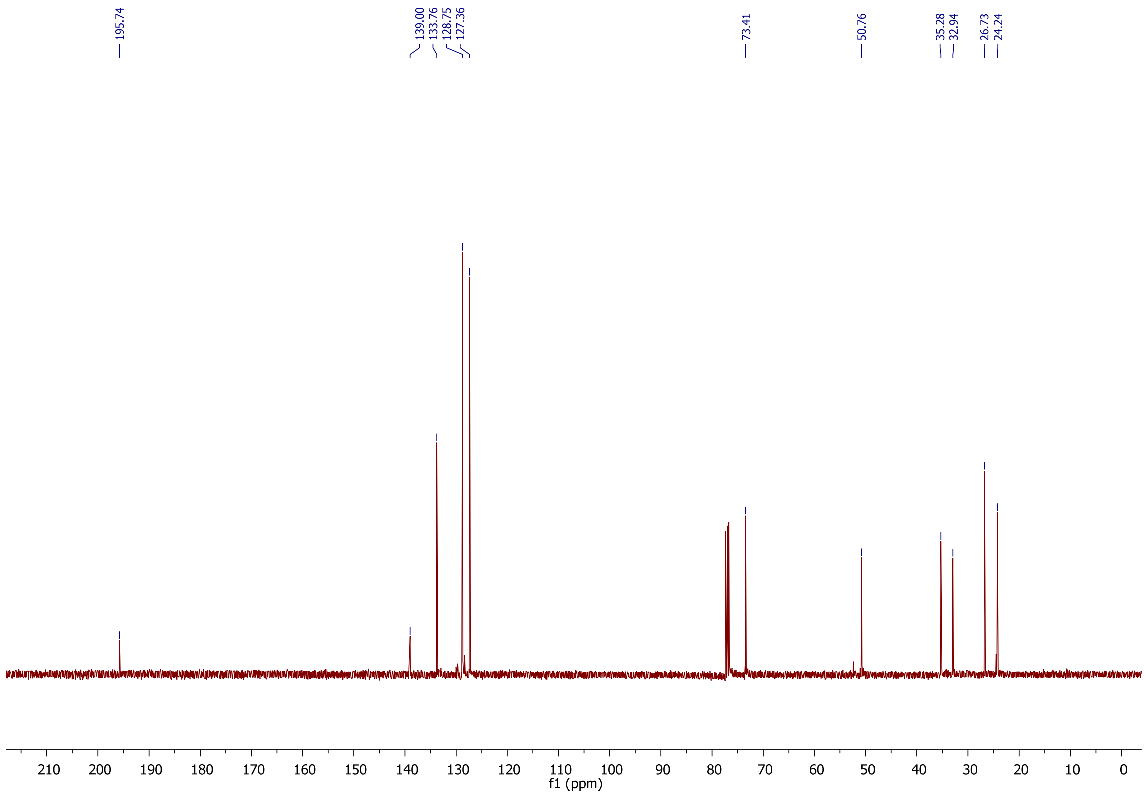

Figure S7. ^{13}C NMR Spectrum of compound **2c** ($CDCl_3$, 100 MHz).

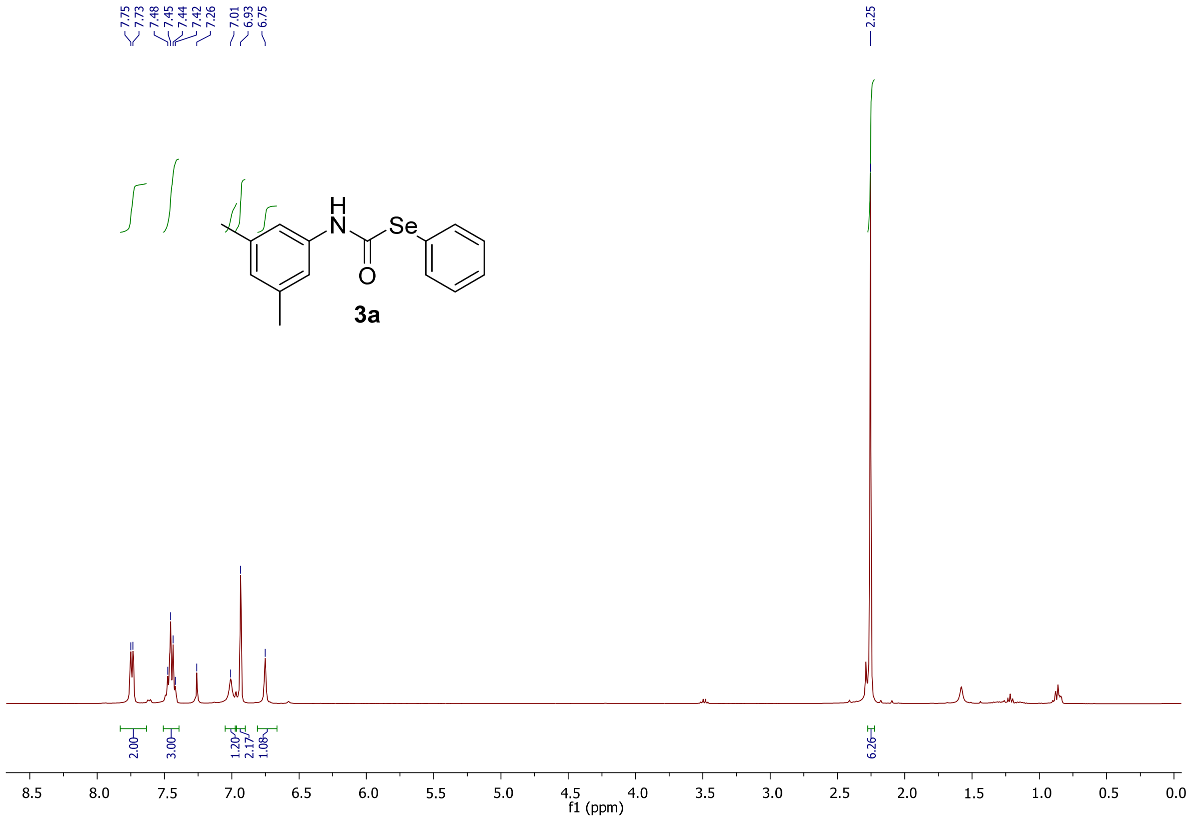

Figure S8. ^{1}H NMR spectrum of compound **3a** ($CDCl_3$, 400 MHz).

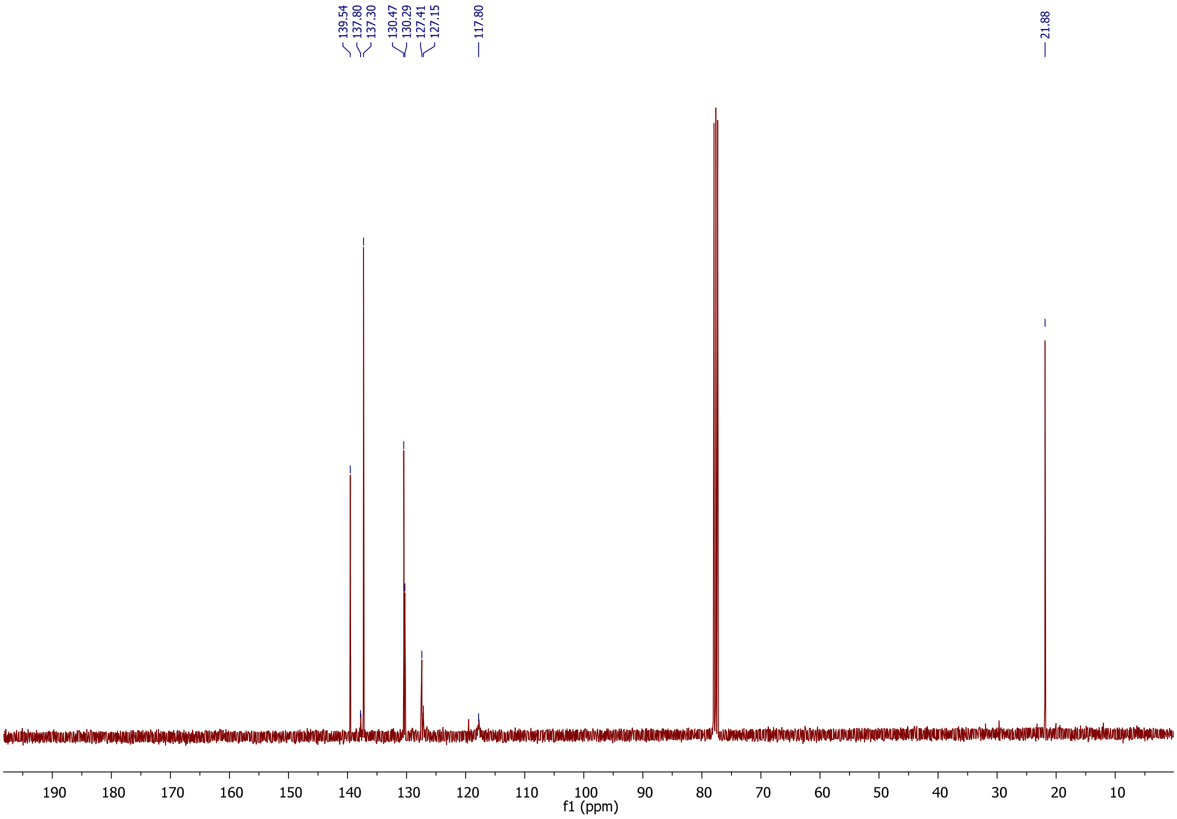

Figure S9. ^{13}C NMR spectrum of compound **3a** ($CDCl_3$, 100 MHz).

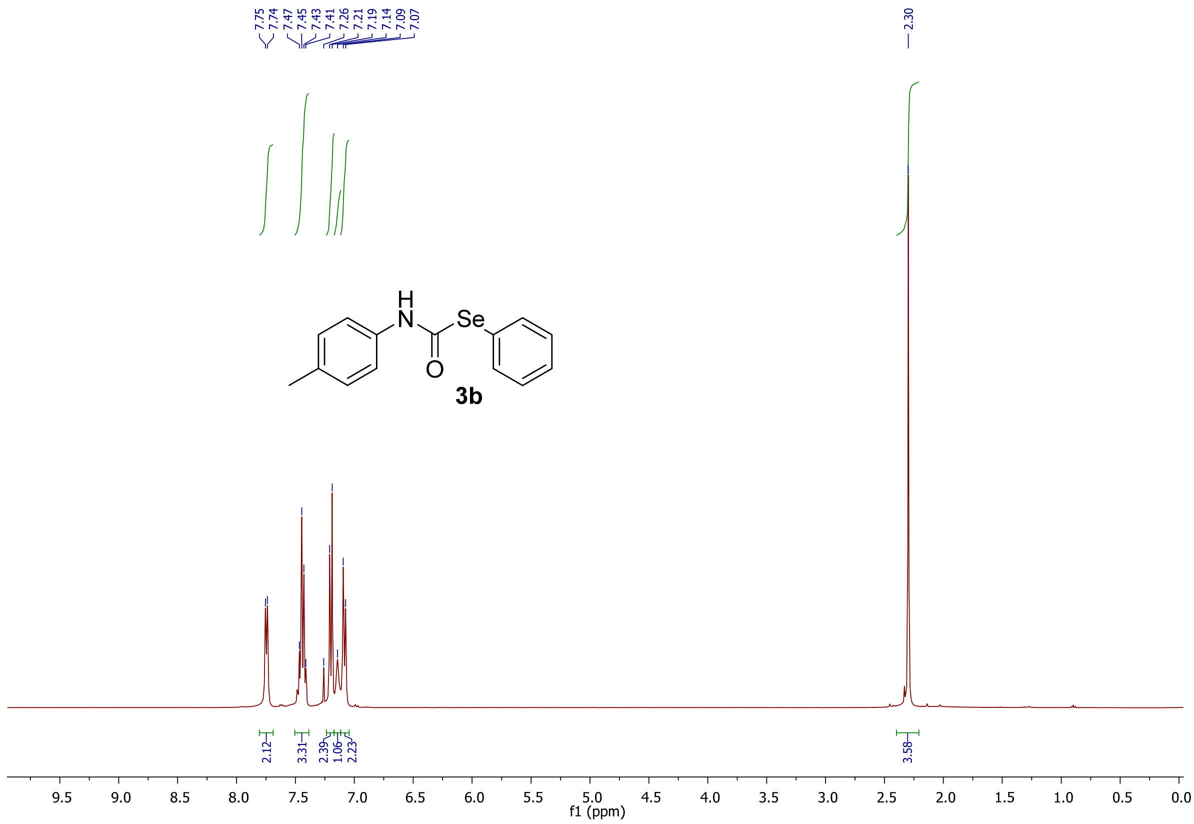

Figure S10. ^{1}H NMR spectrum of compound **3b** ($CDCl_3$, 400 MHz).

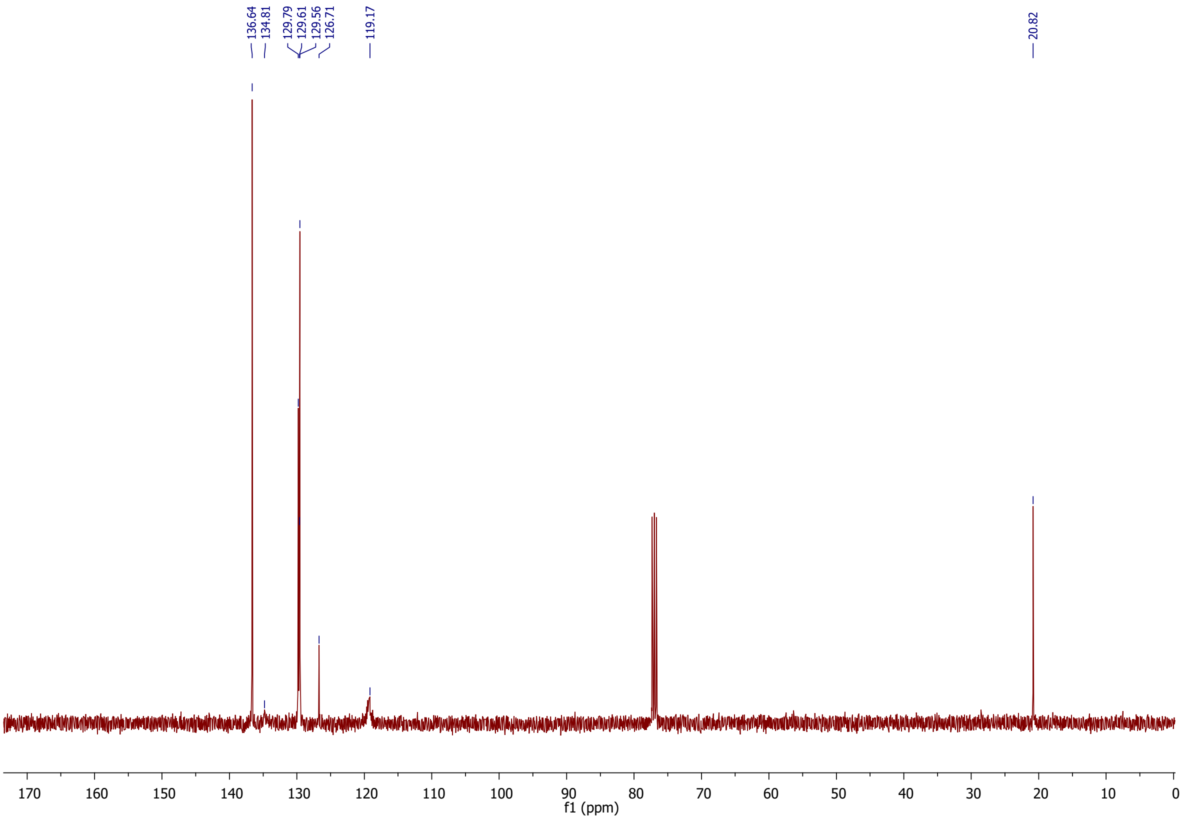

Figure S11. ^{13}C NMR spectrum of compound **3b** ($CDCl_3$, 100 MHz).

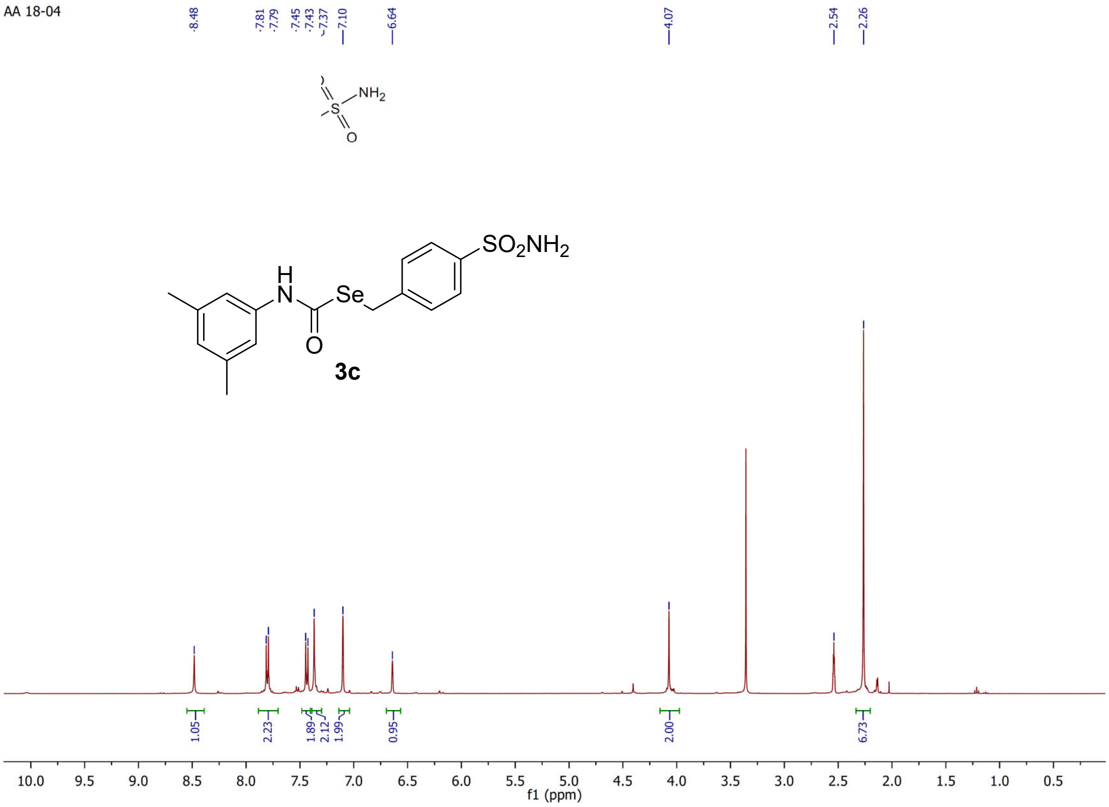

Figure S12. ^{1}H NMR spectrum of compound **3c** (DMSO-d_6, 400 MHz).

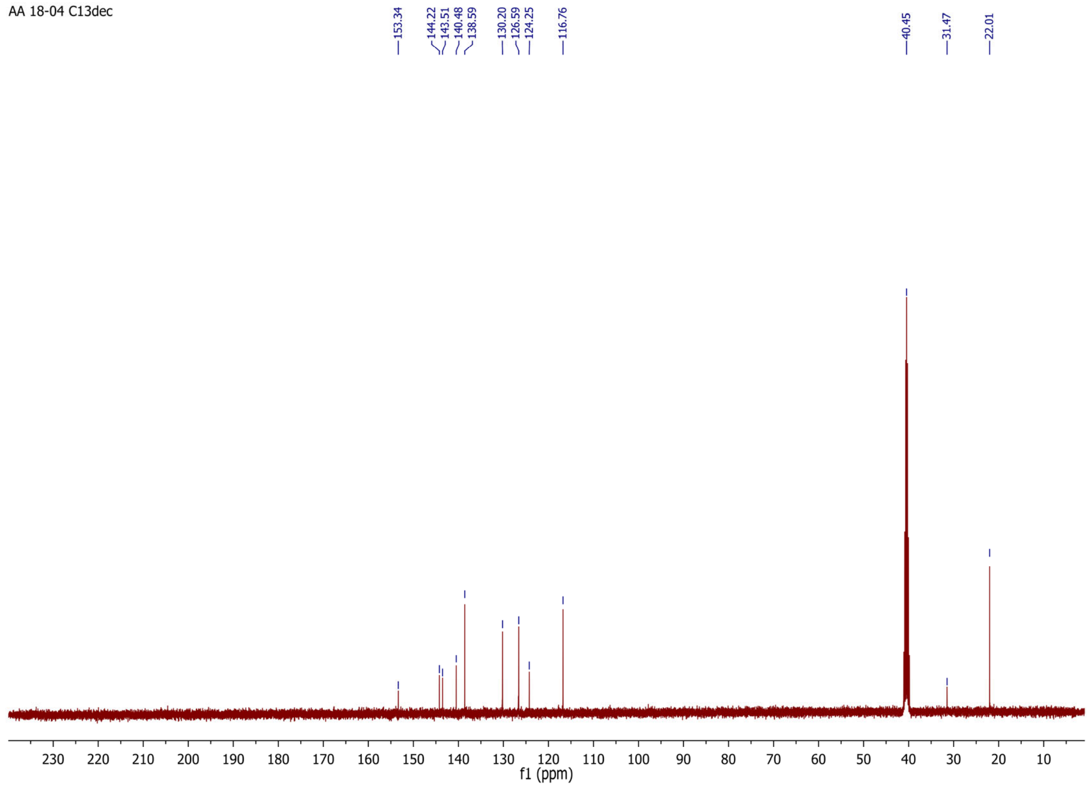

Figure S13. ^{13}C NMR spectrum of compound **3c** (DMSO-d_6, 100 MHz).

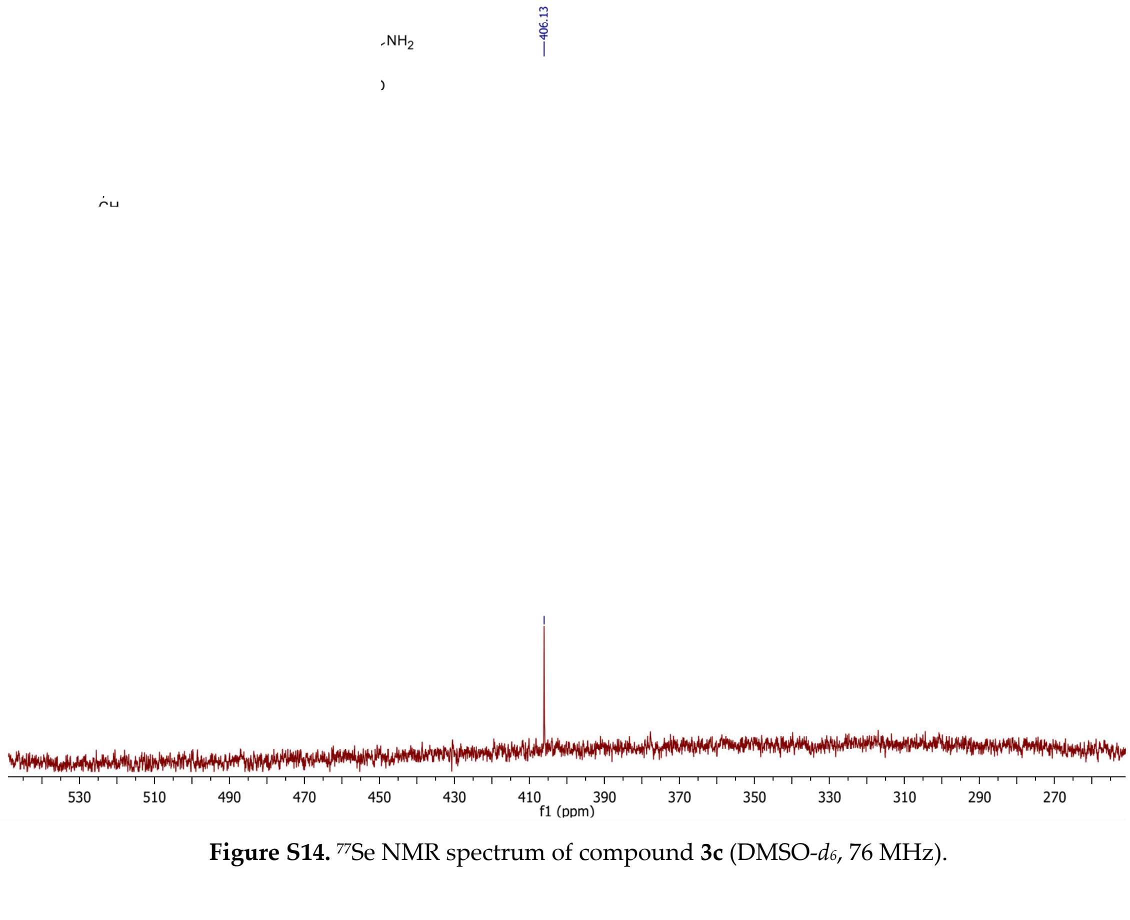

Figure S14. ^{77}Se NMR spectrum of compound **3c** (DMSO-d_6, 76 MHz).

Disclaimer/Publisher's Note: The statements, opinions and data contained in all publications are solely those of the individual author(s) and contributor(s) and not of MDPI and/or the editor(s). MDPI and/or the editor(s) disclaim responsibility for any injury to people or property resulting from any ideas, methods, instructions or products referred to in the content.
